# A Screen for RNA-Binding Proteins in Yeast Indicates Dual Functions for Many Enzymes

**DOI:** 10.1371/journal.pone.0015499

**Published:** 2010-11-11

**Authors:** Tanja Scherrer, Nitish Mittal, Sarath Chandra Janga, André P. Gerber

**Affiliations:** 1 Department of Chemistry and Applied Biosciences, Institute of Pharmaceutical Sciences, ETH Zurich, Zurich, Switzerland; 2 Medical Research Council (MRC) Laboratory of Molecular Biology, Cambridge, United Kingdom; 3 Department of Biotechnology, National Institute of Pharmaceutical Education and Research, Punjab, India; University College London, United Kingdom

## Abstract

Hundreds of RNA-binding proteins (RBPs) control diverse aspects of post-transcriptional gene regulation. To identify novel and unconventional RBPs, we probed high-density protein microarrays with fluorescently labeled RNA and selected 200 proteins that reproducibly interacted with different types of RNA from budding yeast *Saccharomyces cerevisiae*. Surprisingly, more than half of these proteins represent previously known enzymes, many of them acting in metabolism, providing opportunities to directly connect intermediary metabolism with posttranscriptional gene regulation. We mapped the RNA targets for 13 proteins identified in this screen and found that they were associated with distinct groups of mRNAs, some of them coding for functionally related proteins. We also found that overexpression of the enzyme Map1 negatively affects the expression of experimentally defined mRNA targets. Our results suggest that many proteins may associate with mRNAs and possibly control their fates, providing dense connections between different layers of cellular regulation.

## Introduction

Immediately when RNA is synthesized by RNA polymerases, RNA binding proteins (RBPs) assemble on the nascent transcript forming ribonucleoprotein (RNP) complexes, which tightly control all of the further steps in a RNA's life. On one hand, RBPs assist the processing and assembly of non-coding (nc) RNAs into RNP complexes, which mediate essential cellular functions such as splicing and translation [1]. On the other hand, RBPs are essential for mRNA maturation, which involves the addition of a 7-methylguanosine cap at the 5′end of mRNA-precursors, the splicing-out of introns, editing, and the addition of a polyadenosine tail at the 3′end of the message. RBPs further guide mRNA export and localization to specific cytoplasmic loci for translation, and ultimately, they control the decay of (m)RNAs [2]. Notably, all these steps are highly connected to each other and linked with other gene regulatory layers to ensure proper expression of every gene in a cell [3].

The availability of genomic tools now allows the systematic identification of RNA targets for RBPs to obtain a global view of their gene regulatory potential. One of the main approaches include the immunopurification of RNP complexes followed by the analysis of the associated RNAs with DNA microarrays, a method referred to as RNA-immunopurification-microarray (RIP-Chip). Numerous studies applying these genomic tools revealed that many RBPs associate with distinct RNA target sets comprised of a few up to several hundred RNAs, which are often enriched for specific sequence/structural elements that define RBP binding sites. The sets of bound RNAs were often related containing mRNAs coding for functionally or cytotopically related proteins (*e.g.* [4]–[6]; reviewed in [7]–[9]). These findings lead to a model that proposes important coordinative roles for RBPs in the expression of functionally related groups of messages, referred to as ‘RNA regulons’ or ‘post-transcriptional operons’ [7]. Moreover, it underscores that RBPs bind simultaneously and/or sequentially to RNAs generating numerous RNP particles, whose dynamic composition and combinatorial arrangement may be unique for each mRNA expressed in a cell [8]–[10].

RBPs comprise 3 to 11% of the proteomes in bacteria, archaea and eukaryotes underlining the importance of RNA regulation for cell function [11]. In the budding yeast *Saccharomyces cerevisiae*, more than 500 proteins are predicted to function as RBPs [6], [10]. An extensive bioinformatic survey, considering evolutionary conservation, identified almost 100 protein motifs linked to RNA regulation; about half of them have been classified as “enzymatic” domains mostly present in RNA modification enzymes and nucleases. Another 40 motifs or so have been classified as “non-catalytic” RNA-binding domains, which are often part of multi-subunit RNP complexes [11]. Notably, RBPs often contain an array of RNA-binding motifs (RBMs), which further increases the specificity and affinity towards the RNA.

The vast number of protein motifs linked to RNA regulation and the ancient origin of RNA regulation, which is possibly the most evolutionary conserved component of a cell's physiology, proposes that many proteins implicated in other cellular processes could have retained RNA-binding capacity. For instance, several metabolic enzymes in mammals have been shown to bind to and regulate mRNA expression (reviewed in [12]–[15]). Perhaps best characterized are the iron regulatory proteins (IRP), cytoplasmic aconitases that regulate the translation or stability of several messages depending on cellular iron levels [16]. Moreover, a recent comprehensive RIP-Chip study analyzing the RNA targets for more than 40 different RBPs and some other proteins in yeast showed that two metabolic enzymes, for which homologs in mammals have been reported to bind RNA, were reproducibly associated with cellular RNAs, indicating that RNA regulation by these proteins may be evolutionarily conserved [6]. These observations have raised speculations about the existence of yet largely overlooked post-transcriptional regulatory networks between intermediary metabolism and RNA regulation [12]. It furthermore highlights the need of systematic discovery tools to identify novel RBPs as the “universe” of RBPs in eukaryotes could be well underestimated. Possibly many more proteins could have retained or acquired the capacity to bind RNA enabling post-transcriptional gene regulation at yet uncharacterized levels and processes.

In this study, we set out to screen for novel and unconventional RBPs. We therefore used protein microarrays containing 70% of the yeast's proteome and probed them with different sorts of RNA. We selected almost 200 proteins that reproducibly interacted with RNA, most of them not previously annotated to act as RNA-binding proteins such as metabolic enzymes. We further determined *in vivo* associated RNAs for 13 potential RBPs by RIP-Chip. Most of the RBPs bound to distinct subsets of mRNA, some of them code for functionally related proteins and thus, possibly comprise “RNA regulons”. Since this screen is not saturated we expect that many more RBPs - including proteins with dual functions - exist in eukaryotic organisms, forming a dense and robust post-transcriptional scaffold that effectively coordinates gene expression to ensure the integrity and stability of a cells fate.

## Results

### Detection of specific RNA-protein interactions with protein microarrays

**Figure 1:**
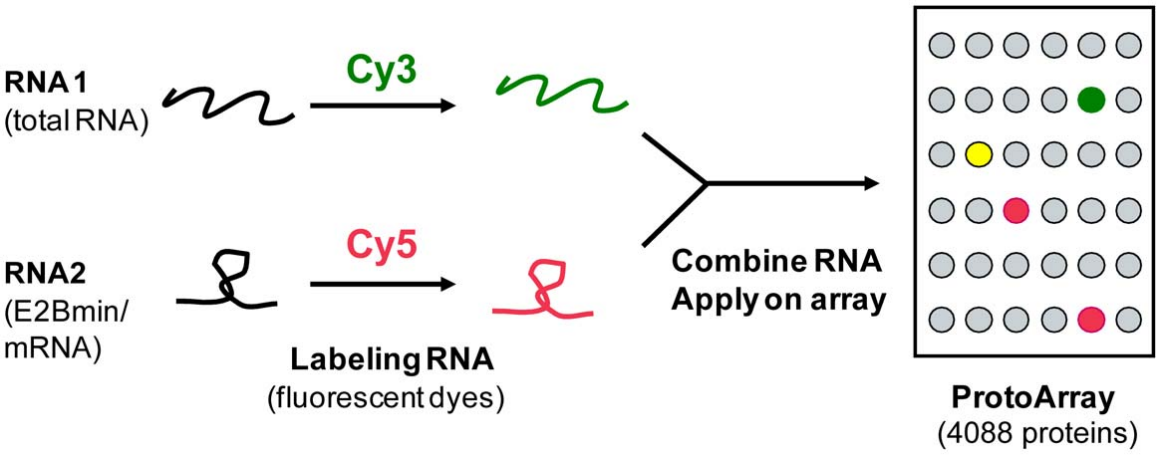
Identification of RNA-binding proteins with protein microarrays. Protein microarrays (Protoarrays) contained 4,088 different yeast proteins (∼70% of the proteome) individually spotted in duplicates onto a modified glass slide. The arrays were probed with a mixture of fluorescently labeled RNAs. After washing, the arrays were scanned and analyzed for proteins that bound either labeled RNAs.

We used functional protein microarrays to screen for proteins that interact with RNA (Figure 1). Protein microarrays have been previously used to identify proteins that interact with small viral RNAs [17], but to our knowledge, there has been no screen to detect proteins interacting with cellular RNAs. To establish the experimental procedure, we first probed protein microarrays with a short 36 nucleotide (nt) long RNA termed E2Bmin, which is a fragment of the Ash1 mRNA known to specifically interact with She2p [18]. She2 is a RBP that facilitates the localization of Ash1 mRNA and other messages to the bud-tip during cell division [19]. Among the 4,088 proteins present on the array, the strongest signal of fluorescently labeled E2Bmin RNA was seen with She2p (24.2 standard deviations [SD] above the mean of signal intensities from two independent experiments; Z-scores are given in Dataset S1). No signals were obtained with an array where proteins were heat-denatured before probing with RNA, indicating that RNA interactions must derive from active proteins. Besides She2, six GTPases (Arf1, Arf3, Arl2, Ypt1, Ypt7, Ypt32; *p*<10^−9^), a tRNA guanylyltransferase (Thg1), and a single-stranded DNA-binding protein (Rim1) also strongly and reproducibly interacted with E2Bmin (SD>3.5 in replicates). Whether these E2Bmin binders may be implicated in the regulation of Ash1 mRNA *in vivo* remains to be elucidated. At least, these experiments show that specific RNA-protein interactions can be detected with our experimental set-up.

### Many enzymes may interact with cellular RNAs

**Figure 2:**
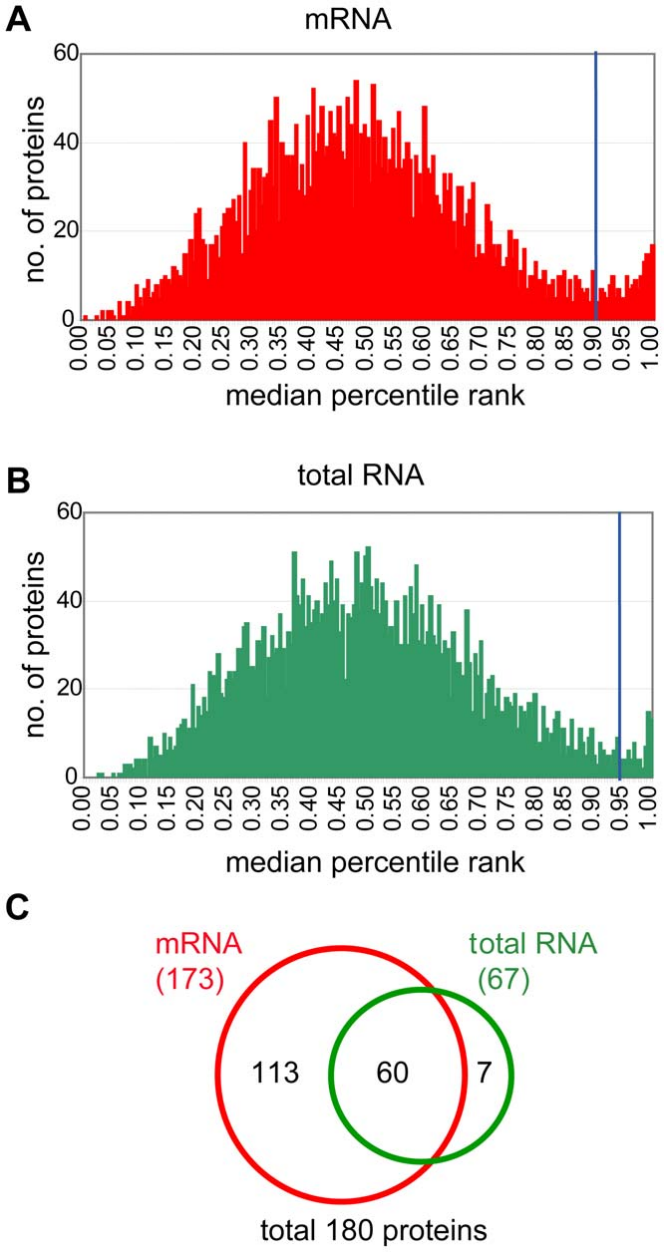
Selection of mRNA and total RNA-binding proteins. (A) Distribution of ranked median signal intensities resulting from protein microarrays probed with mRNAs. The trough at 0.9 was taken as cut-off and all proteins with greater ranks were selected as mRNA binders. (B) Distribution of ranked median signal intensities resulting from arrays probed with total RNA. The trough at 0.95 was taken as cut-off and all proteins with greater ranks were considered as total RNA binders. (C) Venn Diagram representing overlap between proteins binding to total RNA and mRNAs.

To screen for proteins that interact either with total RNA or mRNAs, we basically used the same experimental set-up as applied for the E2Bmin experiments. We probed the protein microarrays with Cy3 labeled ‘total RNA’, which was isolated from yeast cells grown in different carbon sources, and with Cy5 labeled mRNAs isolated from total RNA via oligo-dT columns (see Materials and Methods). Because data was less reproducible compared to the replicate arrays probed with E2Bmin RNA described above, we assigned each element on the array a percentile rank based on background subtracted signals, and calculated median percentile ranks across the five replicates [20] (raw data is provided in Dataset S2). Thereby, the highly ranked proteins represent those with highest signals on the array (e.g. She2p probed with E2Bmin RNA is ranked  = 1 in the above described experiments). The analysis of ranks instead of Z-scores has been previously applied to analyze chromatin immunoprecipitation-chip data and performs well when magnitude and scale of the actual signals varies between replicates [20]. If there are features that are consistently highly ranked across multiple replicates, the distribution of the median percentile ranks of all features will form a bimodal curve; and the median percentile rank at the trough of this bimodal distribution can be selected as a conservative cut-off to define targets [20]. A histogram of the median ranks across the five replicate protein arrays showed a bimodal distribution, which we assumed to represent non-binders and binders, the latter ones to be consistently highly ranked across replicate experiments (Figure 2). We have therefore chosen the trough of the distribution as a conservative cut-off to define proteins that reproducibly interacted with either total RNA or mRNAs; selecting 67 total RNA and 173 mRNA binders, respectively (a list of the total 180 proteins selected from this analysis is provided in Table S1). 90% of total RNA binders were also found in the pool of mRNA binders, but most of the mRNA binders did not strongly bind total RNA (113; 65%). These proteins may preferentially interact with mRNAs, which are underrepresented in the total RNA fraction. However, we wish to note that this selection procedure was designed to go for a robust list of potential RNA binders. It may thus not provide a comprehensive list of all RNA-binders, and further inspection of the data may reveal additional RNA-binders.

**Figure 3:**
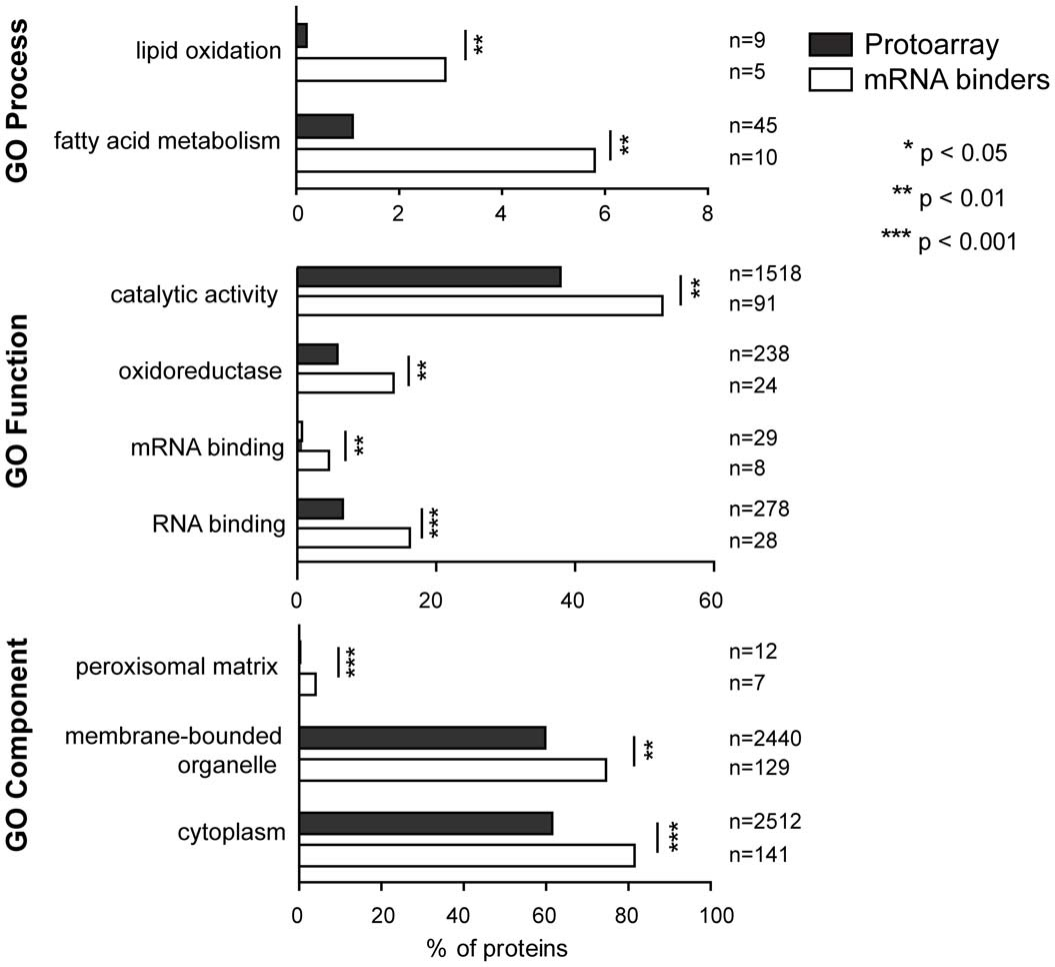
Significantly shared GO terms among mRNA binders. The 173 m RNA binders were searched for significantly enriched GO terms as compared to all the 4,088 proteins present on the protein microarray. Bar diagrams indicate relative amount of genes of the respective GO term among all proteins on the array or among the selected mRNA binders, respectively.

We categorized the selected 180 proteins that either interacted with total RNA or mRNA based on Gene Ontologies (GO) retrieved from the Princeton GO server. 132 out of the 180 proteins (73%) had at least one known function annotated with GO. 28 proteins were annotated with the GO term ‘RNA-binding’, which is therefore over-represented among the group of all 180 selected proteins (*p*<10^−3^, Figure 3; a detailed list of GO terms is provided in Table S2). Further manual inspection of the 180 proteins revealed 18 additional proteins with RNA related functions – adding-up to 46 proteins that act in RNA metabolism (25% of all selected proteins; 35% of proteins with assigned functions; marked in blue in Table S1). In contrast, DNA binding proteins including transcription factors (TFs; 13 proteins, 7%) were not over-represented suggesting that our assay discriminates between DNA and RNA-binders. Moreover, only four of the 180 proteins (Bcy1p, Deg1p, Pfk26p, Yer087p) were among 208 proteins selected in a similar screen applying protein microarrays to identify single- or double-stranded DNA binding proteins [21]. In conclusion, our list of selected proteins bears a substantial fraction of previously known RNA-binders or proteins with RNA-related functions, indicating that our assay likely selected proteins that have RNA-binding properties. However, as outlined above, our stringent cut-off is not expected to identify all of the RNA-binders. Moreover, there are many reasons why diverse known RBPs, which are present on the array did not give reproducible signals across replicates. This includes inactivation of proteins on the slide surface, mis-folding or RNA cross-hybridization in solution, and finally, many annotated RBPs act in protein complexes (e.g. ribosomal proteins) and thus may not specifically interact with RNAs on their own.

Regarding the assigned functions among our list of selected RNA-binders, we were intrigued that many of them have catalytic functions, including oxidoreductases, hydrolases, lyases and transferases (total 94 proteins; 52%, *p*<0.003) (Figure 3, Table S2). Whereas 17 of these enzymes have been previously linked to RNA related processes, the remaining ones act in unrelated processes such as fatty acid metabolism (*p*<0.007) or lipid oxidation (*p*<0.008). Moreover, 25 of these enzymes can be mapped to the yeast metabolic network [22], which are therefore significantly overrepresented compared to all of the metabolic enzymes in this network present on the protein microarray (397 proteins, *p*<0.016). In agreement with this bias for enzymes, most of the herein identified potential RBPs are cytoplasmic (141 proteins, *p*<10^−6^), membrane-associated (*p*<0.003), and some of them located to peroxisomes (*p*<2×10^−5^)(Figure 3). These results indicate that many cytoplasmic enzymes could interact with RNA. In principle, this could provide opportunities to directly connect intermediary metabolism with posttranscriptional gene regulation.

We further analyzed our experimentally defined set of 180 RBPs for the occurrence of protein domains annotated by the Pfam database [23]. 4,049 proteins in *S. cerevisiae* were annotated with 6,119 domains in Pfam, and we analyzed whether some of these domains were over-represented among the 150 proteins (out of 180) that contained at least one Pfam domain (Pfam domains are annotated in Table S1). As expected, most prevalent were known RNA-binding domains such as the K homology (KH_1) domain, the RNA recognition motif (RRM_1) and a subtype of the zinc finger motif, zf-CCHC, which were all significantly enriched (Table 1). Interestingly, several domains were enriched that have not been previously related to RNA function (*p*<10^−3^, hypergeometric) and occur in proteins devoid of other known RNA-binding motifs. This includes the ubiquitin motif or the weakly conserved repeat module PC_rep, which are found in several proteins involved in protein degradation control [24]. It also includes the WW motif and the TPR_1 (tetratricopeptide repeat), which mediate protein-protein interactions and the assembly of multiprotein complexes [25], and several enzymatic domains contained in metabolic enzymes. Whether any of these domains directly or indirectly mediate RNA-binding has yet to be investigated, but their significant overrepresentation makes them prime candidates for further analysis.

**Table 1:** Pfam domains enriched in the list of putative RBPs.

Domain	Occurrence (RBPs)	Occurence (Genome)	Occurrence (Protoarray)	P-value (Hypergeometric)
KH_1	10	18	16	5.44E-09
zf-CCHC	8	23	15	1.06E-06
RRM_1	12	78	41	4.75E-06
ubiquitin	6	15	15	0.00018
PC_rep	4	7	7	0.00048
TPR_1	6	27	19	0.00077
WW	4	8	8	0.00089
adh_short	4	13	9	0.0015
S1	3	6	5	0.0023
TYA	5	81	18	0.0038
Ldh_1_C	2	3	2	0.0041
TBP	2	2	2	0.0041
cNMP_binding	2	5	2	0.0041
Acyl_CoA_thio	2	2	2	0.0041
Ldh_1_N	2	3	2	0.0041
PseudoU_synth_1	2	6	2	0.0041

### Potential RBPs come from different expression regimes

We next asked how the expression of our selected RNA-binders varies across different growth conditions to see whether our selection is biased to any kind of expression characteristics. We therefore compiled a large collection of microarray data available for a wide range of experimental conditions for *S. cerevisiae* from the M3D database [26] (experimental conditions are indicated in the Table S3). Because this data is available in Robust MultiArray (RMA) normalized format [27], it enables direct comparison of expression levels (see Materials and Methods). Expression profiles could be obtained for 164 of the 180 RBPs identified in this study, and we performed K-means clustering with 10 groups to identify subsets of genes that exhibited similar expression patterns. This analysis revealed that the genes followed very heterogeneous expression patterns; genes clustered into different expression regimes namely ubiquitously highly expressed, ubiquitously poorly expressed and specific to conditions (a heatmap cluster of this analysis is shown in the Figure S1).

We further compared the expression levels of the potential RBPs identified in this study with previously annotated RBPs (see Material and Methods). We found no general difference (*p*<0.64, Wilcoxon test). However, our herein identified RBPs are generally higher expressed than non-RBPs (*p*<2×10^−6^, Wilcoxon test); an observation that has been made previously for conventional RBPs as well [28]. We therefore speculate that in particular the highly expressed unconventional RBPs may give good leads for future experiments as they have the potential to control many RNA targets [28].

### Selected novel RBPs associate with distinct sets of mRNAs

To examine whether some novel potential RBPs in our selected list of RNA-binders associate with RNA *in vivo*, we purified endogenously expressed tandem-affinity purification (TAP)-tagged proteins from cells grown in rich media, and identified co-purifying RNAs with yeast DNA oligo arrays. From our list of 180 RBPs, we selected 13 proteins that are expressed at different levels, and for which respective mRNA expression patterns are different across a variety of conditions, providing a representative sample of differentially expressed putative RBPs (marked in Figure S1). Besides a transcriptional regulator (Lap3p) and a co-chaperone (Sti1p), we selected eleven proteins with catalytic activities (Dfr1p, Gre3p, Map1p, Mdh1p, Mdh3p, Meu1p, Pfk2p, Phr1p, Pot1p, Pre10p, Ymr1p), some of them acting in intermediary metabolism, reflecting the fact that many candidate RBPs are enzymes. Seven of the proteins are cytoplasmic, two are peroxisomal, and one representative each are from the nuclear, mitochondrial, ribosomal and proteasome compartment.

**Figure 4:**
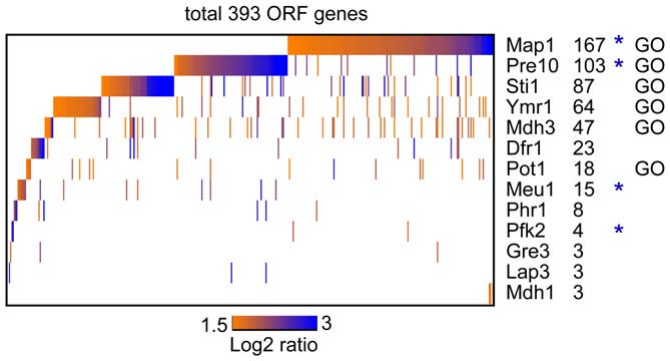
Selected novel RNA-binding proteins bind to distinct sets of mRNAs. (A) Heat map of mRNAs associated with indicated proteins. The color code (orange-blue) indicates the fold-change (log_2_ ratio scale) of the respective feature in the affinity isolation compared to mock control microarray data. The number of mRNA targest for each protein is indicated next to the name of the protein. A star (*) denotes association with own mRNA. ‘GO’ indicates that GO terms are significantly enriched among targets (see Table 2).

We performed three independent affinity isolations with each of the 13 potential RBPs and five independent mock isolations with untagged control cells ( = mock isolates). To identify RNA that were significantly associated with the proteins we selected those features that were on average at least 3-fold enriched in the affinity isolates compared to the mock controls with a *p*-value of less than 0.01 (see Materials and Methods). This analysis revealed that all proteins were associated with unique sets, comprised of a few to dozens of different RNAs (Figure 4; raw data from RIP-Chip experiments and a list of selected features is given in the Dataset S3). Notably, the proteins were almost entirely associated with mRNAs, excluding highly expressed ncRNAs such as rRNAs, tRNAs and snoRNAs. This indicates that these candidate RBPs primarily target mRNAs for potential gene expression control. It also substantiates the specificity of our assays as there is no apparent bias for selection of highly expressed ncRNAs. We also found no correlation between the expression level of these proteins [29] and the number of selected targets (Pearson correlation *r* = 0.04), further substantiating that the observed associations are selective and not merely driven by expression.

Four of the 13 proteins (30%) were associated with their own mRNA (Pfk2, Pre2, Map1, Meu1). Binding to the own mRNA offers the possibility for auto-regulation through the formation of positive or negative feedback loops [30]. This fraction is therefore similar to previous finding with canonical RBPs, where 18 of 46 RBPs (40%) were associated with their own RNA [6]. Remarkably, this fraction is considerably larger compared to TFs, where 10% bound to their own promoter sequences in a global TF-binding site analysis for 106 TFs [30].

Because many RBPs bind to mRNAs coding for functionally related proteins, we searched for common themes among the messages that were associated with the 13 proteins. For six proteins (Map1, Mdh3, Pot1, Pre10, Sti1, and Ymr1) we found significantly enriched GO groups among associated messages, offering the potential to coordinate expression of functionally related groups of messages or ‘RNA regulons’ (Table 2; a more comprehensive list of GO terms is provided in the Table S4). Noteworthy, proteins associated with only a few messages may be less prone for this analysis as the number of the associated mRNAs might be too small to achieve statistically sound data. Although every protein assembled with unique GO terms (e.g. Sti1p bound mRNAs code preferentially for proteins acting in telomere maintenance and DNA recombination), some of the enriched GO terms appeared with more than one protein. For instance, messages associated with Map1p, Pot1p, and Ymr1p are commonly related to translation. However, the particular messages that added to this term were mostly different and only one message (Rps9b) was shared among the targets for the three proteins. Likewise, three proteins (Map1, Mdh3, Sti1) were preferentially associated with messages coding for proteins annotated with pyrophosphatase activity. Among the many targets for these proteins, only eight mRNA targets are shared, which do not link to pyrophosphatase activity (two pyruvate decarboxylases [Pdc1, Pdc5] were commonly enriched; *p*<2×10^−4^). Therefore, it appears that although some GO terms were enriched with more than one of the proteins, it is not because these proteins bound to a common set of messages that connects to one particular GO term, but rather that they were associated with different messages that belong to the same functional class.

**Table 2:** Selected list of GO terms enriched among mRNA targets.

Protein	Category	GO term (p-value)
Map1	Process	translation elongation (10^−7^), small molecule metabolic process (10^−5^)
	Function	catalytic activity (10^−11^), pyrophosphatase activity (2×10^−7^)
	Compartment	plasma membrane enriched fraction (10^−10^), ribosome (4×10^−6^)
Pre10	Function	hydrolase activity (0.007)
Sti1	Process	telomere maintenance via recombination (5×10^−5^)
	Function	helicase activity (10^−14^), pyrophosphatase activity (10^−10^)
Ymr1	Process	translation (3×10^−11^)
	Function	structural constituent of ribosome (10^−10^)
	Compartment	ribosome (6×10^−14^)
Mdh3	Function	nucleoside-triphosphatase activity (10^−3^), helicase activity (2×10^−3^)
Pot1	Process	translation (8×10^−10^)
	Function	structural constituent of ribosome (8×10^−12^)

### Map1p negatively affects gene expression of mRNA targets

**Figure 5:**
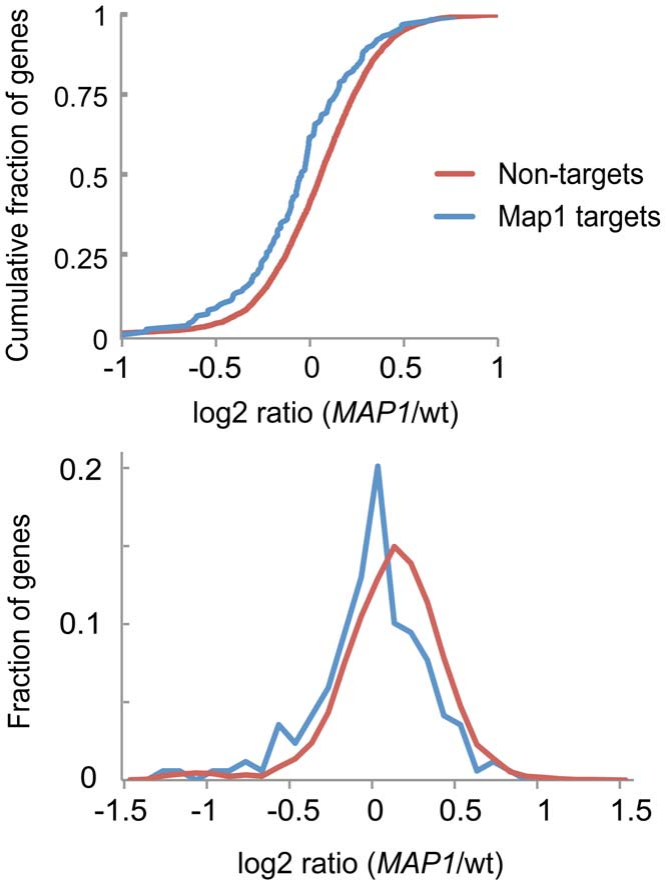
Gene expression profiling of yeast cells overexpressing *MAP1*. Distribution of average Cy5/Cy3 fluorescence ratios from three microarray hybridizations comparing RNA levels of *MAP1* over-expressing yeast cells with control cells. In the upper panel, the fraction of transcripts indicated on the *y*-axis refers to the cumulative fraction of sequences on the microarray; log_2_ ratios are plotted on the *x*-axis. The lower panel shows a histogram depicting the fraction of transcripts (*y*-axis) that are clustred within bins of 0.1 log_2_ ratios (*x*-axis). The red line delineates the distribution of Map1p RNA targets defined from affinity purifications. The blue line represents non-targets.

To investigate how one of the selected candidate enzymes could affect gene expression of targets, we measured the relative changes of mRNA levels of cells overexpressing *MAP1* compared to control cells with DNA microarrays. Map1p is a methionine aminopeptidase (MetAP) that catalyzes the co-translational removal of N-terminal methionine from nascent polypeptides, and it is functionally redundant with Map2p [31], [32]. Notably, Map1p contains two zinc-finger motifs, one CCCC-type and the other of the CCHH-type [33], which occur in DNA-binding proteins and in some RBPs [34] – however these domains were not thought to provide selective RNA-binding but rather to confer interaction of Map1p with the ribosome [35]. Yeast cells bearing a plasmid with *MAP1* under the control of galactose inducible promoter, and control cells containing an empty plasmid, were grown to mid-log phase and expression was induced with 2% galactose for 1.5 hours. Noteworthy, inducible short-time overexpression of RBPs could be beneficial to measure direct effects of proteins on gene expression by minimizing secondary effects that may raise after prolonged alterations of expression levels (Scherrer *et al.*, submitted). We obtained mRNA expression profiles for 6,851 features representing 5,889 yeast genes (raw data is provided in Dataset S4). *MAP1* expression was increased 4.2-fold being the most changed mRNA of all analyzed features. The relative expressions levels of Map1p target mRNAs were very slightly (mean fold change = 0.925) but significantly decreased compared to all non-targets (*p*<10^−5^, Mann-Whitney U test) (Figure 5). Of note, only 44 genes changed at least 1.5 fold with *p*<0.05 (one sample t-test); and seven Map1p targets were overrepresented among the 36 down-regulated messages (*p* = 7×10^−5^, Fisher's exact test). The same analysis with microarray data obtained from cells overexpressing *GIS2* (another ZnF protein among the selected RNA-binders) did not reveal reduced expression of Map1p targets (TS and APG, unpublished results), indicating that the observed shift in the distribution of Map1p targets was not a general effect due to protein overexpression. In conclusion, these results suggest that Map1p could negatively affect mRNA expression of selected mRNA targets.

## Discussion

Protein microarrays have been applied to detect protein-protein, protein-lipid, protein-DNA and protein-viral RNA interactions [17], [21], [36], [37]. Here, we describe the use of protein microarrays for the detection of protein-RNA interactions. We identified dozens of potentially “novel” RBPs that either interacted with mRNA or total RNA on protein microarrays. Strikingly, among these were many enzymes with well-established cellular functions. For some of them, we have shown significant association with functionally related messages, possibly allowing coordination of the expression of ‘RNA regulons’ as seen for *bona fide* RBPs. This was further initially demonstrated for Map1p, for which we observed subtle coordinated down-regulation of target mRNAs upon *MAP1* overexpression, indicating that Map1p preferentially negatively affects gene expression of target messages. This study therefore expands our understanding of post-transcriptional gene networks suggesting regulatory functions to a variety of proteins not connected to gene expression regulation so far.

Our observations that many proteins with enzymatic activities bind to RNAs and potentially participate in RNA regulation are reminiscent to previous observations stating RNA-binding functions for several mammalian enzymes (reviewed in [12], [13]). These mammalian enzymes revealed a striking common denominator – they catalyze reactions that often involve mono- or dinucleotides as substrates or co-factors [14]. Similarly, we found that a large fraction (41 proteins) of the “novel” RBPs with assigned catalytic activities (total 95 proteins) require nucleotide related cofactors/substrates (Table S1): 14 proteins require ATP/AMP as substrate (e.g. kinases), 13 need nicotinamide adenine dinucleotides (NAD) or its 5-phosphate derivate (NADP) as a cofactor, seven employ Coenzyme-A (CoA) found in many enzymes acting in the sterol/fatty acid metabolism, and nine use others such as GTP/GMP or S-adenosyl-methionine. In this regard, the protein binding site for NAD or NADP has been postulated to have occasionally evolved to a binding surface for polyribonucleotides in some mammalian enzymes (e.g. thymidylate synthase (TS) and dihydrofolate reductase (DHFR), as well as glyceraldehyde-3-phosphate, isocitrate, and lactate dehydrogenases) [14]. We tested four (Gre3, Dfr1, Mdh1, Mdh3) NAD binding proteins for association with cellular RNA with RIP-Chip and found that all of them were reproducibly associated with mRNAs, proposing that their NAD binding sites could also have evolved to conduct some RNA regulatory functions. Interestingly, the strong prevalence for nucleotide binding sites among the putatively novel RBPs is also in analogy to recent observations suggesting the existence of transcription regulators that are metabolic enzymes [38]. This raises the possibility that both TFs as well as RBPs might function as direct sensors of the metabolic state of the cell suggesting novel circuits for gene regulation. In this scenario, the binding of metabolic cofactors in the reduced or oxidized form (e.g. NAD/NADH^+^) could differentially regulate the activity of responding RBPs, either through impacting RNA-binding or modulating interaction with other RNP components. Therefore, careful evaluation of the redox state and of the substrate availability will be of further need to decipher the molecular roles of enzymatic RBPs. In addition, modulation of RNA-binding function may result from direct competition between RNA and substrates/cofactors as seen with mammalian IRPs, TS and DHFR [15], [16]. In that case, RNA binding can only occur when substrates are limiting and/or enzymes are in excess and thus, this could possibly contribute to some of the weaker associations seen between RNA and some of the enzyme-related RBPs in our RIP-Chip experiments.

Our screen also proposes RNA binding properties for enzymes that act independently of nucleotides or other cofactors such as peptidases and phosphatases (e.g. Map1, Ymr1). Moreover, our analysis for the enrichment of Pfam domains among our selection of RNA-binders revealed several unexpected domains to be associated with proteins identified in our screen, namely protein-protein interaction domains such as the tetratricopeptide repeat superfamily, which includes the PC_rep and TPR1_domains [23]. We confirmed association of a substantial set of mRNAs with one of the representatives of this family, Sti1p, which contains four TPR1 domains. Although we do not know whether the measured interactions occur directly, it is feasible that some TPR domains could have acquired (or lost) RNA binding functions during evolution: The TPR motif consists of three to 16 tandem-repeats of 35 amino acids that fold into a helix-turn-helix structure and hence, the motif is thought to be closely related to pentatricopeptide repeats (PPR) [39]. The PPR domains rapidly expanded in plants (100–500 genes) where proteins bearing these domains have well established functions in RNA binding, making it reasonable to speculate that some closely related TPR motifs might also have RNA-binding properties.

Nevertheless, the RNA-binding site may also be distinct from the enzymatic site. Diverse examples for shuffling of enzymatic domains next to RNA-binding domains are known such as adenosine deaminases acting on RNA or RNA helicases [40]. Some proteins also retained enzymatic functions in metabolism such as Rib2p in yeast [41]. We analyzed the RNA regulatory potential for Map1p (a methionine aminopeptidase), for which the catalytic domain (peptidase) may be well separated from the RNA-binding sites. The protein contains two Zn-finger domains, which are essential for the normal processing function of MetAP *in vivo* [33] and were thought to provide interaction with the ribosome [35]. However, Zn-finger domains have been widely seen to mediate protein-DNA or protein-RNA interactions [34] and hence, they may act as RNA-binding motifs in Map1p as well. Howsoever, based on your results it appears that Map1p is a dual function enzyme that can negatively affect the expression of some mRNAs targets, including messages coding for proteins that act in translation – in particular translational elongation – and which are therefore in the same process as Map1p.

Several mammalian metabolic enzymes are thought to control the translation or stability of their own mRNAs [13]. For instance, TS binds with high affinity to its own 5′-UTR near the initiator AUG codon and represses translation [15]. Thereby, mRNA binding sites in TS overlap with the binding sites for its substrates, methylenetetrahydrofolate and dUMP and therefore, mRNA and substrate are in direct competition. Likewise, DHFR, a second enzyme in the thymidylate synthesis pathway also binds to its own mRNA, which can be competed by the substrate (folate) antagonist methotrexate [13], [15]. Four proteins, for which mRNA targets were identified with RIP-Chip bound to their own mRNAs, offering the possibility for auto-regulation (Figure 4). Among these was also Pfk2p, which is the β-subunit of the hetero-octameric phosphofructokinase (PFK) involved in glycolysis. Noteworthy, the specific associations of Pfk2p with its own message are independent of the PFK complex, as neither our protein array nor the RIP-Chip analysis revealed significant associations of RNAs with the other subunit of this complex, termed Pfk1p (TS and APG, unpublished results). Since glycolysis is crucial for cell physiology, the activities of enzymes acting in this pathway must be tightly controlled, which is mainly thought being accomplished by transcription and/or the regulation of protein synthesis or degradation [42]. The binding of Pfk2p to its own message could provide an additional layer of expression regulation by controlling the translation, localization or the stability of the message. Such post-transcriptional feedback regulation could add a sensitive mechanism to adapt PFK levels to changing environmental conditions. We wish to note that self-controlling functions among RBPs generally appear to occur more often than among transcription factors, as about 30–40% of RBPs are associated with their own messages compared to 10% of transcription factors that bind to their own promoters [30]. We speculate that such auto-regulation might be beneficial for RBPs in some specific circumstances to control their expression in a temporal and spatial context with respect to other RBPs, and as a means to fine-tune their levels in the cell for appropriate combinatorial interplay.

In conclusion, various instances of enzymes that also act in RNA-metabolism have been previously reported. Our findings put these specific examples into a more general context indicating that RNA regulation by enzymes may be far more common than previously anticipated. A good fraction of (metabolic) enzymes may therefore have a “moonlighting” role in regulating RNA metabolism, which could allow establishing various direct connections between metabolic status and post-transcriptional gene regulation [12]. Future studies on the regulation of mRNA targets by both enzyme-related and conventional RBPs in yeast and other species will help to further shape the RNA-protein interaction network and its regulatory potential and plasticity, and to further establish novel connections between different layers of cellular control.

## Materials and Methods

### Plasmids, strains and media

TAP-tagged strains [29] and the isogenic wild-type strain BY4741 (MAT**a** *his3Δ1 leu2Δ0 met15Δ0 ura3Δ0*), as well as plasmid pBG1805-Map1 [44] were obtained from Open Biosystems. Yeast cells were grown in yeast-peptone-dextrose medium (YPD; 1% yeast extract, 2% bacto-tryptone, 2% glucose) or in synthetic complete medium (SC) [45]. YPGal and YPG are identical to YPD except that they contain 2% galactose or 3% glycerol, respectively, instead of glucose; and SG and SR are identical to SC but contain 2% galactose or 2% raffinose, respectively, instead of glucose. SC-Ura corresponds to SC lacking uracil.

### RNA preparation and labeling

20 pmol of forward and reverse complementary oligonucleotides encoding the E2Bmin sequence [18] and the T7 RNA polymerase promotor were incubated for one minute at 95°C in 20 µl of water and annealed by cooling down the reaction slowly to room temperature (RT). E2Bmin RNA was synthesized by transcription of annealed DNA templates with T7 RNA polymerase (Promega) for two hours at 37°C. The reactions were treated with DNase I (Roche), and RNA was extracted with phenol/chloroform and precipitated with ethanol. The integrity of the RNA fragment was controlled on a 15% polyacrylamide gel containing 8 M urea. Total RNA was isolated from yeast cells by hot phenol extraction [46]. Total RNA was isolated from cells grown either in YPD, SCGal, SD, YPGal, YPG and combined at the ratio (w/w) 2∶2∶1∶1∶1. Messenger RNA was isolated from pooled total RNA with the Oligotex mRNA Mini Kit (Qiagen) according to the manufacturer's protocol. Concentration of RNA was generally assessed by UV-spectrometry with a Nanodrop device (Witeg).

RNA was fluorescently labeled with either Cy3 or Cy5 using the MICROMAX ASAP RNA labeling Kit (Perkin Elmer Cat# MPS544) according to the manufacturer's protocol. Labeled RNA was purified with the RNeasy Micro kit (Qiagen) to remove unincorporated dyes and immediately used for array analysis.

### Protein microarrays and data analysis

We used commercially available protein microarrays containing duplicate probes of 4,088 yeast proteins and additional control proteins spotted on a modified glass slide (ProtoArray™ Yeast Proteome Microarray mg v.1.0; Invitrogen Cat# PA012106; http://www.invitrogen.com). The frozen arrays were thawed at 4°C for 15 min and blocked for 2 hours at 4°C in phosphate-buffered-saline pH 7.4 (PBS; Invitrogen) supplemented with 1% nuclease/protease-free BSA (Equitech-Bio), 1 mM DTT, 50 µg/ml *E. coli* tRNA (Roche), and 50 µg/ml heparin. The arrays were dried by centrifugation at 300 *g* for 1 min at 4°C and immediately probed with fluorescently labeled RNAs. Therefore, Cy3 labeled total RNA (5–10 µg) were combined with either Cy5 labeled mRNAs ( = poly(A)^+^ RNA; 2 µg) or E2Bmin RNA (1.5 µg) and mixed in 60 µl of RNA-binding buffer (RBB, 20 mM Tris-HCl pH 7.9, 75 mM NaCl, 2 mM MgCl_2_, 5% glycerol, 0.05% Triton-X100, 1% BSA, 1 mM DTT, 0.2 mg/ml *E. coli* tRNA, 0.02 mg/ml heparin) supplemented with 6 U of RNaseOUT (Invitrogen, Cat# 10777-019) and applied on the protein microarray, which was covered with a lifterslip (22×60 mm; Erie Scientific). The arrays were put into a sealed hybridization chamber to prevent drying-out, and incubated for 90 min at room temperature in the dark. The slides were washed twice for 10 min at 4°C with 25 ml of RBB buffer supplemented with 10 U/ml RNaseOUT, and twice with 1× RBB buffer lacking tRNA. The arrays were dried by centrifugation at 300 *g* for 5 min and immediately scanned with an Axon Scanner 4200 (Molecular Devices). Data was collected with GenePix Pro 5.1 (Molecular Devices) and imported into Acuity 4.0, which averages data for duplicated spots (Molecular Devices). For data analysis, we removed features representing non-yeast control proteins (e.g. GST) and spots with irregular shapes (FLAG> = 0). Protein microarray raw data have been deposited at ArrayExpress via http://www.ebi.ac.uk/miamexpress/ (accession number: E-MEXP-2897; see below).

To select proteins that bind E2Bmin RNA, we retrieved median signal intensities of background subtracted signals for the red channel (Cy5) probed with E2Bmin RNA. Proteins, for which the signal intensities were at least 3.5 standard deviations (Z score>3.5) above the median of all averaged signals from replicate arrays, were considered as RNA binders (raw data is given in the Dataset S1). To select proteins that interact with total RNA or mRNAs, we retrieved background subtracted median signal intensities of both channels from five replicate arrays, and calculated percentile ranks from 0 to 1 for each channel and array (raw data is given in Dataset S2). The distribution of the median percentile ranks across array replicates was plotted as a histogram and the trough of the bimodal distribution was taken as a conservative cut-off to select proteins that consistently interacted with RNAs (0.90 for mRNA, and at 0.95 for total RNA)(Figure 2).

### RNA affinity isolations

Affinity purification of TAP-tagged proteins was carried out as described previously [4], [6], except that yeast cells were broken mechanically with glass beads in a Tissue Lyser (Qiagen) for 12 min at 300 Hz and 4°C. RNAs from the extract (input) and from the affinity isolates were purified with the RNeasy Mini or Micro Kit (Qiagen), respectively.

### *MAP1* overexpression

100 ml of BY4741 cells bearing plasmid pBG1805-Map1 or the empty plasmid pBG1805 ( = control) were grown in SR-Ura media at 30°C to an OD_600_ of 0.45–0.5 and expression was induced with 2% galactose for 1.5 hours. Cells were generally harvested by centrifugation and washed twice with 800 µl of ice-cold sterile water. RNA was isolated by hot-phenol extraction for microarray analysis as described above [46].

### DNA microarrays and data analysis

70-mer oligo arrays representing features for all annotated nuclear yeast genes (including all ORFs and ncRNAs, introns and some intergenic regions), the mitochondrial genome and various control spots were produced at the Center for Integrative Genomics, University of Lausanne. Arrays were processed and hybridized with fluorescently labeled cDNAs as described previously [47]. For RIP-Chip experiments, 5 µg of total RNA isolated from the extract (input) and up to 50% (∼500 ng) of the affinity purified RNA were reverse transcribed in the presence of 5-(3-aminoallyl)-dUTP and natural dNTPs with a mixture of randome nonamer and dT(20)V primers, and cDNAs were covalently linked to Cy3 and Cy5 NHS-monoesters (GE HealthSciences Cat# RPN5661), respectively, and competitively hybridized on yeast oligo arrays at 42°C for 14 hours in formamide-based hybridization buffer. Gene expression changes upon *MAP1* overexpression were obtained by comparative microarray analysis of Cy3 labeled cDNAs derived from cells expressing the empty vector (pBG1805) and of Cy5 labeled cDNAs from *MAP1* (pBG1805-Map1) expressing cells. Microarrays were scanned with an Axon Scanner 4200A (Molecular Devices) and analyzed with GenePix Pro 5.1 (Molecular Devices). Arrays were deposited and computer normalized at the Stanford Microarray Database [48]. All DNA microarray data are available at the Stanford Microarray Database (SMD) or at the Gene Expression Omnibus at www.ncbi.nlm.nih.gov/geo (accession nos. GSE21850 and GSE21864).

Log_2_ median ratios from three independent RBP affinity isolations and five mock control isolations were retrieved from SMD and exported into Microsoft Excel after filtering for signal over background >1.8 in the channel measuring total RNA derived from the extract. We used the web interface for Cyber-T (http://cybert.microarray.ics.uci.edu/) to employ statistical analyses based on regularized t-tests that use a Bayesian estimate of the variance among gene measurements within an experiment [49]. Features, for which data was obtained in more than 60% of the arrays and that were on average 3-fold enriched with a *p*-value of less than 0.01 in protein affinity isolates compared to mock controls were considered as potential RNA targets (Dataset S3). For *MAP1* overexpression profiling, log_2_ median ratios from three biological replicates were filtered for regression correlation <0.6 and signal over background >2.0 in both channels (Dataset S4).

### Microarray data files

Protein microarray raw data are available at the ArrayExpress database at http://www.ebi.ac.uk/microarray-as/ae/ (acession no. E-MEXP-2897). DNA microarray raw data are available at the Stanford Microarray Database (SMD) or at the Gene Expression Omnibus at www.ncbi.nlm.nih.gov/geo (accession nos. GSE21850 and GSE21864). Microarray data is compliant with MIAME protocol.

### Databases and bioinformatics

Significantly shared GO terms among the selected proteins from the Protoarray screen were identified with the Generic Gene Ontology (GO) Term Finder at the Lewis-Sigler Institute at Princeton University (release 27-Jan-2009; http://go.princeton.edu/cgi-bin/GOTermFinder, [50]) based on annotations in the *Saccharomyces cerevisiae* Genome Database (SGD). Commonly enriched GO terms among mRNAs associated with selected proteins were retrieved with the GO Term Finder that uses a hypergeometric distribution with Multiple Hypothesis Correction to calculate *p*-values (SGD; www.yeastgenome.org). Thereby, we used 6,336 features representing ORF probes for which microarray data was obtained as the background gene set, and only terms with *p*<0.01 (Bonferroni corrected) were considered. Domain annotations for all *S. cerevisiae* proteins were retrieved from the Pfam database (Pfam 24.0) at http://pfam.sanger.ac.uk/ [23]. Significance for enrichment of Pfam domains among RBPs was calculated based on domain content on the Protoarray by using hypergeometric distribution available from the R package for statistical computing.

### Expression analysis of selected RNA-binders across conditions

247 microarray datasets (Affymetrix data) in the form of Robust Multi Array (RMA) normalized profiles were retrieved from the M3D database [26] (conditions are indicated in Table S3). K-means clustering was performed across conditions with the Euclidean distance metric and added into 10 groups. To compare the expression level of novel RBPs against previously documented RBPs [6] and non-RBPs, the latter defined as those which do not encode for documented or novel RBPs, we calculated the median expression level of a gene across the conditions in the M3D dataset and compared the populations using Wilcoxon rank-sum test or Mann-Whitney U test available in the R statistical package [28].

## Supporting Information

Figure S1 **Heatmap of expression profiles for 164 potential RBPs across 247 conditions.** RBPs are clustered with k-means (10 groups) by employing the Euclidean distance as the distance metric to group similarly expressed genes across conditions (see Materials and Methods). Red means high expression and green reflects low expression after the microarray data has been RMA normalized across all experiments. General expression characteristics and the 13 proteins selected for RIP-chip experiments are marked next to the gene cluster. The experimental conditions are described in the Table S3. (TIF)

Table S1 **List of 180 selected proteins interacting with total RNA or mRNA on protein microarrays.** Columns indicate the following (from left to right): YORF, gene name, GO annotations, protein interacted with respective RNA on protein microarrays, classified as metabolic enzyme, number of proteins per cell [28], Pfam domains. Proteins used for affinity isolations are labeled in red. Proteins interacting with mRNAs are marked with red-filled boxes; proteins selected with total RNA are in green; yellow are the ones that interacted with both types of RNA. (XLS)

Table S2 **Significantly enriched GO terms among proteins interacting with total RNA or mRNA.** (XLS)

Table S3 **List of conditions for microarray data retrieved from the M3D database.** (XLS)

Table S4 **A selection of significantly enriched GO terms among messages associated with proteins.** Terms that belong to the GO category ‘Process’ are written in black; GO terms for ‘Function’ are in red, and the ones for ‘Compartment’ are in blue. (XLS)

Dataset S1 **Raw data for protein arrays probed with E2Bmin RNA.** Columns indicate the following (from left to right): YORF, gene name, Z-scores of two protein arrays probed with E2Bmin, mean of Z-scores, Z-scores of heat-treated protein microarray, GO process, function, *S. cerevisiae* Genome Database Identifier. (XLS)

Dataset S2 **Raw data of protein arrays probed with total RNA and mRNA.** Background substracted median fluorescent signals are shown for both channel, and the percentile ranks and selected proteins preferentially interacting either with mRNA or total RNA are also shown. A key describes different worksheets. (XLS)

Dataset S3 **RIP-Chip data for 13 potential RBPs.** Features/ORFs that were at least 3-fold enriched compared to mock isolates with *p*<0.01 are indicated in a separate worksheets. Therein, fold changes (log_2_ ratios) are in black, *p*-values are shown in red. (XLS)

Dataset S4 **Microarray raw data of** ***MAP1*** **overexpressing compared to control cells.** Columns indicate the following (from left to right): Spot ID (SMD); YORF; Gene name; log_2_ ratio for triplicate experiments (MAP1/control); average log_2_ ratio; average fold-change; standard deviation; *p*-value; Map1 target (1 = target, 0 = non-target). (XLS)
