# Supplementary figures and images for: A Screen for RNA-Binding Proteins in Yeast Indicates Dual Functions for Many Enzymes
Source: PLoS One. 2010 Nov 11;5(11):e15499. doi: 10.1371/journal.pone.0015499 (PMC2988813; doi:10.1371/journal.pone.0015499)

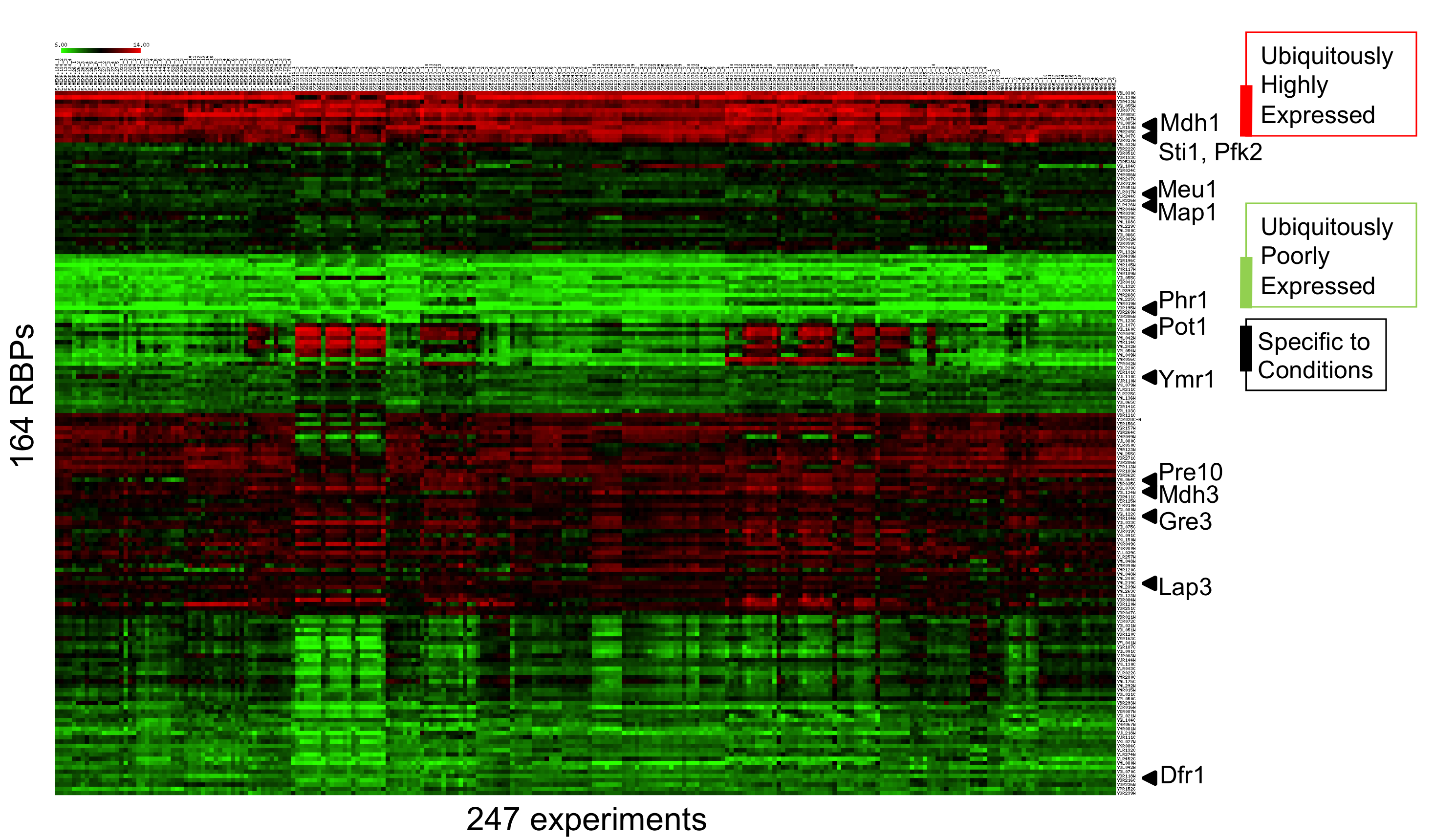

Supplement: Figure S1 — Heatmap of expression profiles for 164 potential RBPs across 247 conditions. RBPs are clustered with k-means (10 groups) by employing the Euclidean distance as the distance metric to group similarly expressed genes across conditions (see Materials and Methods). Red means high expression and green reflects low expression after the microarray data has been RMA normalized across all experiments. General expression characteristics and the 13 proteins selected for RIP-chip experiments are marked next to the gene cluster. The experimental conditions are described in the Table S3. (TIF) [file pone.0015499.s001.tif]
